# A *Promotora de Salud* Model for Addressing Cardiovascular Disease Risk Factors in the US-Mexico Border Region

**Published:** 2008-12-15

**Authors:** Héctor Balcázar, Matilde Alvarado, Robert Fulwood, Veronica Pedregon, Frank Cantu

**Affiliations:** University of Texas Health Science Center at Houston, School of Public Health, El Paso Regional Campus; National Heart, Lung, and Blood Institute, National Institutes of Health, Bethesda, Maryland; National Heart, Lung, and Blood Institute, National Institutes of Health, Bethesda, Maryland; University of California, San Francisco; Health Resources and Services Administration, Rockville, Maryland

## Abstract

**Introduction:**

In 2002, the National Heart, Lung, and Blood Institute partnered with the Health Resources and Services Administration's (HRSA*'*s) Bureau of Primary Health Care and Office of Rural Health Policy to address cardiovascular health in the US-Mexico border region. From 2003 through 2005, the 2 agencies agreed to conduct an intervention program using *Salud para su Corazón* with *promotores de salud* (community health workers) in high-risk Hispanic communities served by community health centers (CHCs) in the border region to reduce risk factors and improve health behaviors.

**Methods:**

*Promotores de salud* from each CHC delivered lessons from the curriculum *Your Heart, Your Life*. Four centers implemented a 1-group pretest-posttest study design. Educational sessions were delivered for 2 to 3 months. To test *Salud para su Corazón*-HRSA health objectives, the CHCs conducted the program and assessed behavioral and clinical outcomes at baseline, 3 months, 6 months, and 12 months after the intervention. A 2-sample paired *t* test and analyses of variance were used to evaluate differences from baseline to postintervention.

**Results:**

Changes in heart-healthy behaviors were observed, as they have been in previous *Salud para su Corazón* studies, lending credibility to the effectiveness of a *promotores de salud* program in a clinical setting. Positive changes were also observed in low-density lipoprotein cholesterol level, triglyceride level, waist circumference, diastolic blood pressure, weight, and glycated hemoglobin (HbA1c).

**Conclusion:**

Results suggest that integrating *promotores de salud *into clinical practices is a promising strategy for culturally competent and effective service delivery. *Promotores de salud* build coalitions and partnerships in the community. The *Salud para su Corazón-*HRSA initiative was successful in helping to develop an infrastructure to support a *promotores de salud* workforce in the US-Mexico border region.

## Introduction

Since 1990, cardiovascular disease (CVD) has been the leading cause of death among the US Hispanic population (1). An estimated 23.8% of all Hispanic deaths in the United States in 2002 were due to diseases of the heart. According to the Centers for Disease Control and Prevention (CDC), the highest concentration of heart disease death from 1996 to 2000 was found among Hispanics living in the border region (2). The extent of disease has prompted a focus on cardiovascular health as an area for intervention among communities in the border region.

In 2002, the National Heart, Lung, and Blood Institute (NHLBI) partnered with the Health Resources and Services Administration's (HRSA*'*s) Bureau of Primary Health Care and Office of Rural Health Policy to address cardiovascular health in the US-Mexico border region. The NHLBI-HRSA interagency relationship resulted in an agreement from 2003 through 2005 to conduct an intervention using the cardiovascular health promotion program *Salud para su Corazón* (Health for Your Heart) with *promotores de salud* (community health workers) in 4 high-risk Hispanic communities in the border region (3-6). By implementing the program in community health centers (CHCs), the *Salud para su Corazón*-HRSA initiative provided the opportunity to test the effectiveness of *Salud para su Corazón* as a community-based clinical program during a 3-year period, yielding clinical outcome data for the first time.

The purpose of this article is 3-fold: 1) to describe the strategies used by the NHLBI-HRSA partnership with 4 HRSA-funded CHCs to implement cardiovascular health promotion and disease prevention activities in their respective communities; 2) to describe the effects of *Salud para su Corazón* interventions on behavioral and clinical outcomes; and 3) to describe the lessons learned during implementation and evaluation of *Salud para su Corazón* in all 4 health care settings.

All 4 participating CHCs provide primary health care and intervention services to predominantly Hispanic patient populations. They were chosen to conduct the *Salud para su Corazón *program because of their location in the US-Mexico border region, the array of settings for primary health care and intervention that they presented, and the high CVD death rates of the communities they served (Table 1). They included Centro San Vicente (CSV) in El Paso, Texas; Gateway Community Health Center, Inc, (GCHS) in Laredo, Texas; North County Health Services (NCHS) in San Marcos, California; and Mariposa Community Health Center (MCHC) in Nogales, Arizona.

CSV is a nonprofit health care and social services agency that has served the El Paso community for more than 15 years. CSV comprises 3 clinic sites throughout the region with more than 13,000 registered patients. Sixty-eight percent of the patients earn less than the federal poverty threshold; 97% are Hispanic; 74% are best served in Spanish.

GCHC is a nonprofit health care corporation that has been operating in Laredo for 42 years. GCHC has 2 clinics, serving approximately 15,000 residents annually. More than 32% of Laredo's population falls below the federal poverty threshold. Of GCHC's patient base, 95% are Hispanic and 61% do not have health insurance.

NCHS is a nonprofit health care corporation operating in underserved areas of San Marcos for the past 32 years. NCHS comprises 9 stationary clinics and 1 mobile clinic. The service area covers approximately 57,000 people, many of whom are newly arrived immigrants. Seventy percent of the patients are Hispanic; the average patient does not have health insurance and has not obtained education past a sixth-grade level. NCHS is the only CHC that does not have paid *promotores* on staff.

MCHC is a nonprofit health care corporation that has served Nogales for more than 22 years. MCHC has 2 sites, 1 of which is dedicated to health promotion and disease prevention education. The center serves approximately 18,000 patients annually. More than 30% of pediatric patients come from low-income families, and more than 90% of the residents are Hispanic. Approximately 40% of the patients are younger than 20 years.

## Methods

### Implementation strategies

The following primary objectives were shared by all participating CHCs: 1) increase CVD knowledge and heart-healthy practices; 2) increase participation in physical activity; 3) decrease blood pressure, cholesterol levels, blood glucose levels, and body mass index (BMI); 4) increase awareness and knowledge through community-based health promotion activities; and 5) increase involvement and support for *Salud para su Corazón *from local agencies and organizations. To meet these objectives, all 4 CHCs implemented *Salud para su Corazón *by identifying community strengths and appropriately adapting methods employed by previous programs for use in their respective communities (3-6).

GCHC uses several components within the center to integrate self-management interventions into the center's medical practice. Center components include patients, *promotores*, medical providers, certified diabetes educators, medical support staff, administrators, and the board of directors. The training for *Salud para su Corazón *is integrated into the medical providers' referral system after the patient is referred to the *promotores*.

The clinical directives for implementing the *promotores* model at GCHC can be summarized as follows: 1) community outreach by *promotores* facilitates patient appointments with the medical staff (physician visit) facilitates patient appointments with the medical staff (physician visit), and appointments are scheduled; 2) an assessment plan is developed by the medical staff; 3) medical staff initiate educational directives (issuing verbal and printed instruction); 4) a treatment plan is developed that includes laboratory assessment, medication, care plan, and referral to the *promotores* program; 5) the *promotores* program includes group classes (*Su* *Corazón*,* Su Vida* [*Your Heart, Your Life*] supported by the *Salud para su Corazón*-HRSA program) and individual support; and 6) follow-up is provided by both medical and *promotores* staff as needed.

### Community participation and capacity building

The *Salud para su Corazón*-HRSA initiative adopted several approaches to community participation and capacity building. It established collaboration among select staff from NHLBI and HRSA and researchers from the University of Texas School of Public Health, El Paso Regional Campus, to provide consultation, mentorship, and guidance to CHC staff, *promotores*, and other personnel involved with project activities at the community level. This team delivered *Salud para su Corazón *resources and materials to all 4 CHCs to support delivery of community education. For example, it conducted monthly calls for the duration of the project with key staff and lead *promotores* from each CHC to review progress and to provide an opportunity to discuss any aspect of the projects.

### Train-the-trainer activities

All of the CHCs conducted a series of well-defined and structured *promotores* training activities, following protocols similar to those previously implemented in other *Salud para su Corazón* programs (3-6). These training protocols consisted of approximately 16 to 18 hours of training to complete the *Your Heart, Your Life* curriculum lessons. Lead *promotores* who had been previously trained in the *Your Heart, Your Life* manual delivered the training activities to *promotores* new to the *Salud para su Corazón *program. Lead *promotores* from all 4 CHCs provided leadership and served as role models and mentors for the newly trained *promotores*.

### Delivery of *Your Heart, Your Life* program activities

*Promotores de salud* from each CHC delivered 8 lessons from *Your Heart, Your Life,* which uses various educational approaches and relevant materials. More information about the curriculum can be found elsewhere (3-7). Table 2 provides an overview of the health education program at each site. All 4 centers implemented a 1-group, pretest-posttest study design. *Promotores'* primary responsibility was to recruit Hispanic participants through the CHCs*'* clinical and community outreach systems. Recruitment strategies included referrals by the medical team, advertisement of the program at the CHCs, and outreach to neighborhood and community sites near the CHCs. Educational sessions were delivered several times a week, once a week, or every other week for a total intervention period of 2 to 3 months.

### Media, community outreach, and partnerships

As part of program activities for the *Salud para su Corazón*-HRSA initiative, each CHC conducted media and community outreach events to enhance the work of the *promotores *at the community level (3,8). CHCs organized different activities to enrich the programs in their communities. In addition to media and community outreach events, the CHCs developed partnerships with various local health clinics, health departments, schools, and community-based organizations to support program activities.

### Evaluation of change in behavior and clinical outcomes

We conducted an evaluation workshop in El Paso to present CHC principles and strategies for data collection and to integrate an evaluation component into the *Salud para su Corazón*-HRSA program. To test whether the program met its health objectives, the CHCs agreed to conduct the program and to assess behavioral and clinical outcomes at 4 intervals: at baseline and at 3 months, 6 months, and 12 months after the intervention. Actual data collection varied among sites (Table 2). Clinical measures included weight, BMI, low-density lipoprotein (LDL) cholesterol level, high-density lipoprotein (HDL) cholesterol level, triglyceride level, glycated hemoglobin (HbA1c), and systolic and diastolic blood pressure. Behavioral data were collected by using My Family Habits Scale, a 35-item, self-reported, valid and reliable instrument, which has been tested in previous *Salud para su Corazón promotores *programs (3-6). NCHS also included an assessment of physical activity. Behavioral data using My Family Habits Scale are presented for NCHS only (behavioral data from other sites were not available). The main objective of the evaluation was to examine changes in clinical measures from baseline to 3 different points after delivery of the intervention (3, 6, and 12 months) because the effect of *Salud para su Corazón* on clinical outcomes had not been explored previously.

### Statistical analyses

Data on sociodemographic characteristics were not collected uniformly across sites and therefore were not included in the statistical analyses. For the purposes of evaluation, we combined clinical data from 2 CHCs (CSV and GCHC) because of their similar timing of assessments and similar integration of clinical and community outreach activities that used *promotores*. Behavioral and clinical data for NCHS are presented separately. Data from MCHC, which assessed outcomes after 2 months of follow-up, did not yield significant changes in clinical outcomes and are not presented here.

We used a 2-sample paired *t* test to evaluate differences from baseline to 6 months  and from baseline to 12 months  for CSV and GCHC. Repeated-measures 1-way analyses of variance were used to evaluate matched sample comparisons between baseline and 2 follow-up points (3 and 6 months after the intervention) for NCHS.

## Results

We found statistically significant decreases from baseline to 6 months after the intervention for 3 clinical outcomes: diastolic blood pressure, LDL cholesterol level, and HbA1c (Table 3). Only LDL cholesterol level and triglyceride level showed significant decreases from baseline to 12 months after the intervention. Mean BMI did not change and remained in the obese category at 12 months for participants from both CHCs (Table 4).

We also noted improvements in behavioral and clinical data from NCHS. Significant improvements in heart-healthy behaviors were observed for the 3 subscales of My Family Habits. Participants increased the frequency of reporting their consumption of healthy amounts of salt and sodium, cholesterol, and fat, and engaging in behaviors related to healthy eating for adequate weight. We also observed significant changes in waist circumference and weight. Waist size decreased from 37.4 inches at baseline to 36.1 inches at 3 months after the intervention and 36.14 inches at 6 months after the intervention. After 3 months of follow-up, study participants' weight had also decreased and was maintained after 6 months of follow-up. The proportion of study participants who reported engaging in physical activity after 3 months of follow-up showed a significant increase from baseline; the change observed at 3 months of follow-up was maintained after 6 months of follow-up.

## Discussion

The *Salud para su Corazón*-HRSA initiative achieved positive changes in heart-healthy behaviors and improved the CVD risk profile among most study participants. It also succeeded at capacity building and infrastructure development.

### Behavioral and clinical outcomes

The improvements in heart-healthy behaviors observed by NCHS were consistently documented in previous *Salud para su Corazón *studies, lending credibility to the efficacy of the *promotores* program in a clinical setting (3-6). The positive changes further observed in some clinical outcomes (eg, LDL cholesterol level, triglyceride level, waist circumference, diastolic blood pressure, weight, and HbA1c) suggest that integrating community outreach with a clinical protocol that links medical diagnosis, referrals, and community health group education is a promising strategy for culturally competent and effective health service delivery (9,10).

Limitations in design and methods temper interpretation of results. Additional empirical testing with more integrated and sophisticated intervention approaches is needed (10). Nevertheless, both behavioral and clinical outcomes provided the impetus for investing in a *promotores* infrastructure at the clinics to address CVD health disparities among Hispanics (11). We hope to achieve consistency in research methods and to integrate evaluation protocols as part of the delivery of health care and intervention services in the region.

### Capacity building and infrastructure development

The investment in *promotores *is a step toward building community coalitions and partnerships to support work in various sectors of the health care system (12). The *Salud para su Corazón*-HRSA initiative succeeded in taking the first step toward developing an infrastructure to support the *promotores* workforce in the US-Mexico border region.

One key element of the NHLBI-HRSA initiative was pilot testing several integrated clinic-type models of care that link *promotores* to the medical system at the clinical level. The GCHC illustrates the integration of *promotores* made possible through the support of the initiative.

Several key components of success of this clinical *promotores* integration include 1) open and frequent communication; 2) wide organizational acceptance of *promotores;* 3) regular staff meetings (in which *promotores* participate) to assess progress and identify issues; 4) extensive training of *promotores;* 5) thorough documentation; 6) management support; 7) provider involvement including training, recruitment, support, and participation; and 8) regular assessment of patient satisfaction and feedback. Finally, the GCHC has identified several benefits of its *promotores* program. The benefits to the provider include more efficient use of time, reinforcement of the treatment plan, assessment of social needs and concerns, extension of physician services, identification of health advocate and additional clinic and service referrals, and improved patient control of diabetes. Benefits to the patient include more time dedicated to education, increased awareness of the need to adhere to treatment plans, individualized care, improved access to care, specific needs met by appropriate referrals, and improved health outcomes.

### Limitations

The study has limitations related to research design and to methods of data collection and evaluation. The *Salud para su Corazón*-HRSA initiative was not able to implement a more sophisticated study design. This limitation is faced by many community-based organizations, including CHCs, because of the great need to provide direct community outreach and clinical services to their clients. Additionally, CHCs did not collect sociodemographic and other pertinent information that may confound intervention effects because of resource limitations (ie, not having personnel at the CHCs to support data collection). As a result, less emphasis and priority were given to research and to adequate collection of data to support the CHCs' *Salud para su Corazón*-HRSA programs.

Challenges still exist in implementing standardized research protocols. Data collection tools were difficult to standardize. We were unable to consolidate the development of a complete database that could match each participant with all data points needed for all variables of interest (ie, age, sex, socioeconomic status, acculturation status, marital status, and educational level). As a consequence of this limitation, the evaluation is constrained by the difficulty in conducting analyses of confounding factors. Therefore, these results should be interpreted with caution. Expertise in evaluation and statistical analyses needs to be an intricate part of the infrastructure being developed for CHCs as community outreach activities are integrated with clinical encounters. The implementation of electronic records appears to be a promising strategy.

Finding the right balance between allowing flexibility with the design and the intervention (including data collection procedures) and infusing a well-developed, science-based approach is a major challenge in these types of health promotion and disease prevention initiatives. Community-based participatory research is a promising approach that needs to be strengthened with CHCs when *promotores* are working in conjunction with the medical system to combat heart disease and address CVD risk factors among Hispanic populations living in the US-Mexico border region (13).

### Recommendations

We offer recommendations based on the experience of the *Salud para su Corazón*-HRSA initiative to enhance the capacity of CHCs and their providers to ensure that the *promotores* model can be sustained in their community settings.

- **Include support groups.** Complement the *Salud para su Corazón* training with informal support groups. The benefits of support groups are to help participants maintain healthy behaviors and to facilitate follow-up with participants.
- **Provide a clinical measures graph. **Use the clinical measures to help motivate participants. Provide a graph of the key measures to give participants a visual image of their progress.
- **Develop a referral, follow-up, and documentation system.** Produce an effective system that is consistent with self-management clinical practice. Include referral, follow-up, feedback, and documentation practices to standardize and track participant and program information.
- **Extend the evaluation timeline.** Measures taken at baseline and 6 and 12 months after the intervention are crucial to documenting a program's success. Projects should be given at least 1 year to evaluate their outcomes.
- **Provide evaluation training.** Evaluation training is needed for program coordinators and* promotores*. Without critical evaluation skills, the programs are unable to adequately assess their respective programs and provide justification for additional funding.

## Figures and Tables

**Table 1:** Characteristics of Community Health Centers, Including County and State Age-Adjusted Cardiovascular Disease (CVD) Death Rates of Hispanics Aged ≥35 Years, 1996-2000^a^

Community Health Center	Location	Setting	CVD Death Rates^b^		

			County	State	National
Centro San Vicente	El Paso, TX	Community education	465	412	348
		Clinic-based education			
		Paid *promotores de salud*			
Gateway Community Health Center, Inc	Laredo, TX	Clinic-based education	458		
		Paid *promotores de salud*			
North County Health Services	San Marcos, CA	Community education	329	339	
		Volunteer *promotores de salud*			
Mariposa Community Health Center	Nogales, AZ	Community education	401, X County 405, Y County	401	
		Paid *promotores de salud*			

a Rates per 100,000 and spatially smoothed.

b Source: Centers for Disease Control and Prevention (2).

**Table 2:** Program Overview, *Salud para su Corazón*, 2003-2005

**Component**	**Mariposa Community Health Center**	**North County Health Services**	**Centro San Vicente**	**Gateway Community Health Center, Inc**
Study design	Pre/post; convenience sample	Pre/post; convenience sample	Pre/post; convenience sample	Pre/post; convenience sample
Measurement interval	Baseline, 2 months	Baseline, 3 months, 6 months	Baseline, 3 months, 6 months, 12 months	Baseline, 6 months, 12 months
Total participants recruited	37	106	22	91
No. of community health workers trained during 3-year period^a^	57	16	73	28
Method of delivery	Group education	Group education	Group education	Group education

a Not all participated in the program.

**Table 3:** Baseline to Posttest (6 months) Differences for Study Participants of *Salud para su Corazón*, Centro San Vicente and Gateway Community Health Center, Inc (n = 85^a^), 2003-2005

Variable	Mean (SD)		*t* Test^b^	*P* Value

	Baseline	Posttest		
Weight, lbs	182 (40)	183 (39)	0.73	.46
BMI, kg/m^2^	33 (8)	33 (7)	0.32	.75
Systolic blood pressure, mm Hg	129 (17)	130 (15)	0.13	.89
Diastolic blood pressure, mm Hg	77 (10)	74 (10)	2.61	.01
LDL cholesterol, mg/dL	108 (34)	95 (32)	3.88	<.001
HDL cholesterol, mg/dL	48 (12)	48 (12)	0.66	.51
Triglyceride level, mg/dL	178 (77)	170 (76)	0.92	.35
HbA1c, %	8 (2)	7 (1)	3.65	<.001

Abbreviations: Lbs, pounds; BMI, body mass index; LDL, low-density lipoprotein; HDL, high-density lipoprotein; HbA1c, hemoglobin A1c (glycated hemoglobin).

a Total n is different from value shown in Table 2 of 113 for both community health centers because of missing responses. Posttest evaluations were conducted 6 months after baseline measurements.

b Two sample paired *t* test with 84 degrees of freedom for each *t* statistic shown.

**Table 4:** Baseline to Posttest (12 months) Differences for Study Participants of *Salud para su Corazón*, Centro San Vicente and Gateway Community Health Center, Inc (n = 85^a^), 2003-2005

**Variable**	Mean (SD)		***t* Test^b^**	***P* Value**

	**Baseline**	**Posttest**		
Weight, lbs	182 (40)	179 (40)	0.49	.62
BMI, kg/m^2^	33 (8)	32 (7)	0.68	.50
Systolic blood pressure, mm Hg	129 (17)	127 (16)	1.15	.25
Diastolic blood pressure, mm Hg	77 (10)	82 (17)	0.60	.54
LDL cholesterol, mg/dL	108 (34)	86 (27)	4.71	<.001
HDL cholesterol, mg/dL	48 (12)	49 (12)	0.20	.84
Triglyceride level, mg/dL	178 (77)	155 (70)	2.27	.02
HbA1c, %	8 (2)	8 (7)	0.05	.96

Abbreviations: Lbs, pounds; BMI, body mass index; LDL, low-density lipoprotein; HDL, high-density lipoprotein; HbA1c, hemoglobin A1c (glycated hemoglobin).

a Total n is different from value shown in Table 2 of 113 for both community health centers because of missing responses. Posttest evaluations were conducted 12 months after baseline measurements.

b Two-sample paired *t* test with 84 degrees of freedom for each *t* statistic shown.
